# Are comparable studies really comparable? Suggestions from a problem-solving experiment on urban and rural great tits

**DOI:** 10.1007/s10071-024-01885-3

**Published:** 2024-07-09

**Authors:** Ernő Vincze, Ineta Kačergytė, Juliane Gaviraghi Mussoi, Utku Urhan, Anders Brodin

**Affiliations:** 1 Department of Biology, Lund University, Lund, S-223 62 Sweden; 2 HUN-REN – PE Evolutionary Ecology Research Group, Centre for Natural Sciences, University of Pannonia, Veszprém, Hungary; 3 Department of Ecology, Swedish University of Agricultural Sciences, Box 7044, Uppsala, 750 07 Sweden; 4 School of Biological Sciences, University of Auckland, Auckland, New Zealand; 5 Netherlands Institute of Ecology (NIOO-KNAW), Wageningen, The Netherlands

**Keywords:** Cognitive ability, Urban and rural environment, String-pulling, Plug-opening, Experimental replicability

## Abstract

Performance in tests of various cognitive abilities has often been compared, both within and between species. In intraspecific comparisons, habitat effects on cognition has been a popular topic, frequently with an underlying assumption that urban animals should perform better than their rural conspecifics. In this study, we tested problem-solving ability in great tits *Parus major*, in a string-pulling and a plug-opening test. Our aim was to compare performance between urban and rural great tits, and to compare their performance with previously published problem solving studies. Our great tits perfomed better in string-pulling than their conspecifics in previous studies (solving success: 54%), and better than their close relative, the mountain chickadee *Poecile gambeli*, in the plug-opening test (solving success: 70%). Solving latency became shorter over four repeated sessions, indicating learning abilities, and showed among-individual correlation between the two tests. However, the solving ability did not differ between habitat types in either test. Somewhat unexpectedly, we found marked differences between study years even though we tried to keep conditions identical. These were probably due to small changes to the experimental protocol between years, for example the unavoidable changes of observers and changes in the size and material of test devices. This has an important implication: if small changes in an otherwise identical set-up can have strong effects, meaningful comparisons of cognitive performance between different labs must be extremely hard. In a wider perspective this highlights the replicability problem often present in animal behaviour studies.

**Supplementary Information:**

The online version contains supplementary material available at 10.1007/s10071-024-01885-3.

## Introduction

Replicability and generalizability are two important measures of the validity of scientific studies. While conceptual replications, i.e. studies investigating the same hypothesis with different methodology, are relatively common, direct replications of scientific experiments are rare (Brecht et al. 2021; Farrar et al. 2021). Both types of replications are used for looking at the generalizability of the results and for comparisons within and between species (Kabadayi et al. 2016, 2017; Isaksson et al. 2018; Urhan et al. 2023). However, the validity of such generalizations and comparisons is often questionable, considering that replications can be challenging: even if experimenters try to create as identical lab conditions as possible, it is very likely that there will be differences between different set-ups. Even in intraspecific, but especially in interspecific comparisons, most animals have been tested in different labs by different experimenters and with different apparatuses. In the study we are presenting in this paper, testing problem-solving abilities in great tits (*Parus major*), we have found unexpected differences between study years and observers, which has drawn our attention to this problem, as relatively minor and apparently non-instrumental changes to the experimental set-up seemed to cause large differences in the responses of the tested birds.

The cognitive ability we tested was innovativeness, defined as the ability to solve new problems or to find new solutions to old problems and to remember these solutions so that they can be used to exploit new resources (Reader and Laland 2003). Innovation per se is very hard to study as it happens very infrequently. When a new foraging behaviour spreads in a population through social learning, the occasion when an individual first used it, i.e. the innovation, will rarely be known. Instead, the common way to test innovativeness is to present animals with problem-solving tasks they have not encountered before (Cole et al. 2011; Griffin and Guez 2014). In such studies, animals are typically required to solve a task in order to get a reward, frequently consisting of some desirable food (Benson-Amram and Holekamp 2012; Griffin and Guez 2014). However, solving success in these tasks may be affected by other characteristics besides innovativeness, such as experience, current motivational state, and other cognitive traits (Rowe and Healy 2014; Horik and Madden 2016). Furthermore, solving success may depend on experimental design; therefore, designing tests that adequately estimate innovativeness in animals in a standardised manner is a challenging task. Additionally, comparing the change in solving success and latency over repeated sessions can also be a test of individual learning ability: increasing success and/or decreasing latency may indicate that the tested animals are learning from experience (Boogert et al. 2006; Morand-Ferron et al. 2011).

Although most studies of avian cognition have been conducted in corvids and large parrots (Lambert et al. 2019), an increasing body of evidence suggests that many smaller passerines are also quite cognitively capable, with parids (tits, titmice and chickadees) being particularly good at solving cognitive challenges (Sasvári 1979; Isaksson et al. 2018; Audet et al. 2023). Within the parid family, the great tit seems to outperform its relatives in cognitive tasks and learning (Sasvári 1979; Exernová et al. 2006; Johnsson and Brodin 2019; Urhan et al. 2023). In nature, the great tit is a generalist species known for its innovative foraging behaviour (Overington et al. 2009; Morand-Ferron et al. 2011; Johnsson and Brodin 2019), which may facilitate good problem-solving abilities.

One very well-known cognition test that has been performed on many different animal species is known as the string-pulling test. In this test, a reward, typically food, is attached to a string. In order to get access to the reward, the animal has to pull the string. In tests with birds, the string is typically hanging vertically in a position where the attached food is directly inaccessible to the bird. To reach the reward the bird has to hold the pulled-in loops of the string so that the reward does not fall down out of reach again (Jacobs and Osvath 2015). Before our experiment, there were three studies where wild great tits have been brought into the lab and tested in cages in string-pulling experiments (Thorpe 1956; Vince 1956; Cole et al. 2011). However, solving success in these studies varied greatly, to an extent that it is less likely to be due to natural variation between populations, and more likely due to differences in methodology such as the reward and the experimental regime.

The original primary aim of our study was to test for differences between urban and rural birds, as it is not well understood how urbanization affects cognitive performance. It has been suggested that urban populations should perform better than rural ones, because innovativeness can be beneficial both for colonizing a novel habitat (Sol et al. 2002) and for exploiting novel, anthropogenic resources (Rodewald et al. 2011). Despite this, previous studies on various species yielded mixed results (Griffin et al. 2017; Lee and Thornton 2021; Vincze and Kovács 2022). The great tit is common in its original forest habitat as well as a successful colonizer of anthropogenic habitats such as city parks and suburban gardens. In this species, two studies demonstrated that birds in more urbanized habitats tend to perform better in problem-solving tasks compared to their less urbanized conspecifics (Preiszner et al. 2017; Grunst et al. 2020). However, both of these studies were performed on breeding great tits at their nests in the wild, where environmental factors like microclimate and food ability, which may differ between urban and rural habitats and may affect problem-solving success, cannot be controlled. Therefore, it is not well-known how well urban and rural great tits perform in problem-solving tasks outside the breeding season, under indoor conditions where environmental factors can be controlled and standardized.

We aimed to test the following questions with the study: (i) how do great tits perform in the string-pulling test compared to earlier studies (Thorpe 1956; Vince 1956; Cole et al. 2011)? (ii) How do great tits perform in another problem-solving task, a plug-opening test, compared to their American relative, the mountain chickadee *Poecile gambeli* (Kozlovsky et al. 2015, 2017)? (iii) Do the birds show a learning effect in the above two tasks, i.e. does their problem-solving performance improve over repeated sessions? (iv) Is there a relationship between the performance in the two tasks? And most importantly: (v) is there a difference between the problem-solving performance between urban and rural great tits under controlled captive conditions? We also discuss the unexpected differences between study years, which was included in our models as a control variable.

## Methods

### Subjects and housing

We captured great tits in three urban areas (a city park in Malmö, 55.6001° 12.9899°, population density in 2020: 4 150 people//km^2^; and two sites in Lund, 55.7144° 13.2069° and 55.6976° 13.2472°, population density in 2020: 3 535 people/km^2^, source: https://www.citypopulation.de/en/sweden/cities/) and eight rural sites (seven within 10 km from the town of Höör, 55.9346° 13.5278°, and one at Stensoffa in the Svedala region, 55.6947° 13.4494°; population density: <5 people/km^2^) in Scania, Southernmost Sweden (TableS1), using mist nets set up next to bird feeders that we previously set up. The Malmö and Lund sites consisted of an urban matrix of large buildings surrounded by major roads, pedestrian walkways, and lawns interspersed with a mix of native and non-native tree species (for details on species composition in Malmö, see Jensen et al. 2022). The rural sites were in forested areas, with no active farms or inhabited houses near the capture locations. The most common trees in these forest habitats were common oak (*Quercus robur*), lime (*Tilia cordata*), elm (*Ulmus glabra*), birch (*Betula sp*.), Norwegian spruce (*Picea abies*) and hazel (*Corylus avellana*).

We captured and tested 20 birds in 2015 (September to December) and 10 birds in 2016–2017 (December to February). As the results were inconclusive, we resumed the experiment by capturing and testing 36 additional birds in 2021–2022 (September to February), resulting in a total sample of 66 birds. Between these years, there were several, mostly unintentional minor differences in the experimental methods (summarized in Table 1), which we elaborate at each respective part of the description of our methods. We did not capture birds from March to late August when great tits are breeding and moulting. After capture, we marked each bird with one unique numbered metal ring as well as one or two plastic colour rings for visual identification in the lab. We used plumage characteristics to age and sex them. We then transported the birds in individual cotton bags to an indoor animal facility at the Department of Biology, Lund University. The transport took a maximum of 30 min.

**Table 1:** Summary of methodological differences between the four years. Sample sizes are the total number of captured birds in a given year, followed by the numbers of urban males (UM), urban females (UF), rural males (RM) and rural females (RF). The numbers after the site names indicate the number of birds captured from that site in a given year. See also Table S1 for the numbers of each age and sex group in each site

Year	2015	2016–2017	2021	2022
Experimenter	A	B	C	C
Sample size	**20** (5 UM, 5 UF, 5 RM, 5 RF)	**10** (2 UM, 2 UF, 4 RM, 2 RF)	**24** (7 UM, 5 UF, 7 RM, 5 RF)	**12** (2 UM, 4 UF, 2 RM, 4 RF)
Urban sites	Ekologihuset (7), Malmö (3)	Ekologihuset (4)	Ekologihuset (6), Linero (6)	Linero (6)
Rural sites	Backen (3), Orups sjukhus (3), Växsjön (2), Stensoffa (2)	Gäddangen (2), Karlsund (1), Linekulsvägen (2), Växsjön (1)	Gäddangen (6), Ormapumpan (6)	Karlsund (3), Linekulsvägen (3)
Period	September to December	December to March	September to December	January to February
Neophobia dish size	Small (35 mm)	Small (35 mm)	Large (85 mm)	Large (85 mm)
Inter-trial intervals	24 to 48 h	1 to 288 h	24 to 48 h	24 to 48 h
Test order	Always string first	Random	Varied among birds (but not within)	Varied among birds (but not within)
Other perches	Removed	Not removed	Not removed	Removed
String cover tube material	Thin plastic	Thin plastic	Sturdy plastic	Sturdy plastic, thin rim
Plugged tube size and material	Small (75 × 11 mm) glass	Small (75 × 11 mm) glass	Large (100 × 15 mm) plastic	Small (75 × 11 mm) glass
Plugged tube place	High (41 cm)	Low (26 cm)	Low (26 cm)	High (41 cm)
Worms	2	1	1	1

We housed the birds in individual 55 × 56 × 36 cm cages that we had positioned on shelves in an enclosed compartment along a wall in the room. The cages were placed two by two so that each bird had visual contact with one neighbour. The room had lighting with an outdoor light spectrum and computer-controlled light and temperature regimes. In mornings and evenings, an automatic one-hour dimming function simulated dawn and dusk, following outdoor day length patterns. We kept the temperature constant at 14 °C, which is a temperature that works well in this type of experiment (Brodin and Urhan 2014, 2015; Isaksson et al. 2018). Before we started any training or experimental sessions, we allowed the birds to get accustomed to the environment in the lab for at least two days (i.e. we started the tests no earlier than the morning of their third day in captivity), which is sufficient according to our experiences from previous studies (Brodin and Urhan 2014, 2015).

The birds had *ad libitum* access to a food mixture of seeds and nuts, a suet cake and water that was changed daily. The water was enriched with a commercial vitamin supplement for birds. We cleaned the cages every day. Before each testing session, we visually inspected the birds and made sure that they were in good condition. We avoided handling the birds during the experimental sessions to minimise stress. When we had finished all experimental sessions on a bird, we released it at the same location as it was originally captured, 10 to 26 (mean ± SD = 17.8 ± 4.6) days after capture. Before we released a bird, we checked whether it was in adequate body condition (i.e. no injuries or feather damage and sufficient fat reserve). The study complies with Swedish and EU animal welfare legislations and regulations.

### Neophobia test

Animals may frequently be wary of new and unknown objects, a phenomenon known as neophobia, causing them to avoid novel objects. Hence, there is a risk that an animal’s inability to solve a task may depend on neophobia towards experimental objects rather than an inability to pass the test (Greenberg 2003; Audet et al. 2016). To control for this, we performed a neophobia test, two to five days after capture in 2015 and 2021–2022, and following another experiment in 2016–2017 (Isaksson et al. 2018). The test was performed on the birds in their home cages, visually separated from their neighbours. We started the test with a control stage in which we presented a mealworm (*Tenebrio molitor*) on a ceramic dish (diameter: 10 cm) that the birds had been familiarized with before the test. We repeated this procedure five times with each bird to control for within-individual variation in feeding latencies. If the bird refused to take the mealworm for over 30 min in this control stage, we terminated the experiment and repeated it the next day. All but one bird (an urban adult female from 2021) took the five mealworms in the control stage in either the first or the second neophobia test. As we could not calculate a neophobia score for this one bird, it is excluded from models in which neophobia is included as a covariate (see Statistical analyses).

After the fifth session, we presented the mealworm on an unfamiliar plastic plate (diameter: 35 mm in 2015–2017, 85 mm in 2021–2022) that was painted with broad red and green stripes, placed on top of the ceramic plate. Such a plate with a striking novel colouration should be a good reason for neophobic behaviour to manifest. The most common neophobic action is that it takes a longer time to approach the novel plate than the familiar one. We observed the bird until it consumed the worm from the coloured, novel, plate. There was one bird (an urban adult male, also from 2021) that consumed all five mealworms in the control stage but did not consume the worm in the neophobia stage for 30 min; we terminated the test for this bird and considered its latency to be the maximum allowed time, 1800 s. We calculated neophobia in the same way as Audet et al. (2016), as the difference in seconds between when the bird took the mealworm from the new brightly coloured plate and the old, non-painted plate. For the latter, we used the mean of the five sessions in the control stage. We then log-transformed the neophobia score (adding 400 s to all data points so that we get positive values for all of them) to get the variable closer to a normal distribution.

### General experimental protocol

Following the neophobia test, each bird participated in two types of problem-solving tests: the string-pulling test and the plug-opening test. Each of the two tests was repeated four times for each bird. These test sessions were 20 min long, and (except for 2016–2017) were always performed in pairs where one type of test immediately followed the other. For all birds in 2015 and half of the birds in 2021–2022, the string-pulling test always preceded the plug-opening; for the other half of the birds in 2021–2022, the order was reversed, i.e. the plug-opening always preceded the string-pulling, to investigate a possible effect of test order. The four pairs of sessions were performed at least 24 but no more than 48 h after one another. In 2016–2017, the birds participated in the same number of plug-opening and string-pulling tests (4 each) as in the other years, but the test regime was more irregular: several test sessions of one or both types could be performed on the same day; and there could be several days long gaps (up to 12 days) between two sessions of the same type. In spite of these irregularities in the experimental regime, the 10 birds in this group showed similar learning patterns to the 56 birds with stricter regime (see Results), so we opted to include them in our models.

At the start of the experimental sessions, the focal bird in its home cage was visually (but not acoustically) isolated from the other birds by moving the cage to a separate shelf (2015–2017) or a desk in the same room (2021–2022) and closing off the housing compartment. After turning off all lights, the observer removed all food from the cage and set up the experimental device for the first test. The lights were turned off so that the bird could not see the device getting set up. In 2015 and 2022, the perches, except for the one next to the test device, were also removed from the cage; in 2016–2017 and 2021, all perches were kept in the cage. Following this, the observer moved to a booth covered by dark, one-way glass to get out of the bird’s sight and turned on the lights for the bird. After the bird had solved the task, or succeeded to eat the worm by other means (see below), or lost the worm by dropping it where it could not reach it, or after the maximum time of 20 min, the observer turned off the lights again, replaced the device for the first test with the device for the second test, and repeated the above protocol. All sessions were video recorded. Regardless of whether or not a bird solved a problem in the first session, we performed four experimental sessions on all birds to test whether their solving performance improved with each repeat, indicating learning within task type. All neophobia and problem-solving sessions were recorded on camera (type: Toshiba Camileo S20); however, out of the 516 problem-solving sessions, 66 recordings are not available due to technical malfunctions during recording or file saving. See Online Resource 1 for a sample of these videos.

### String-pulling

**Fig. 1:**
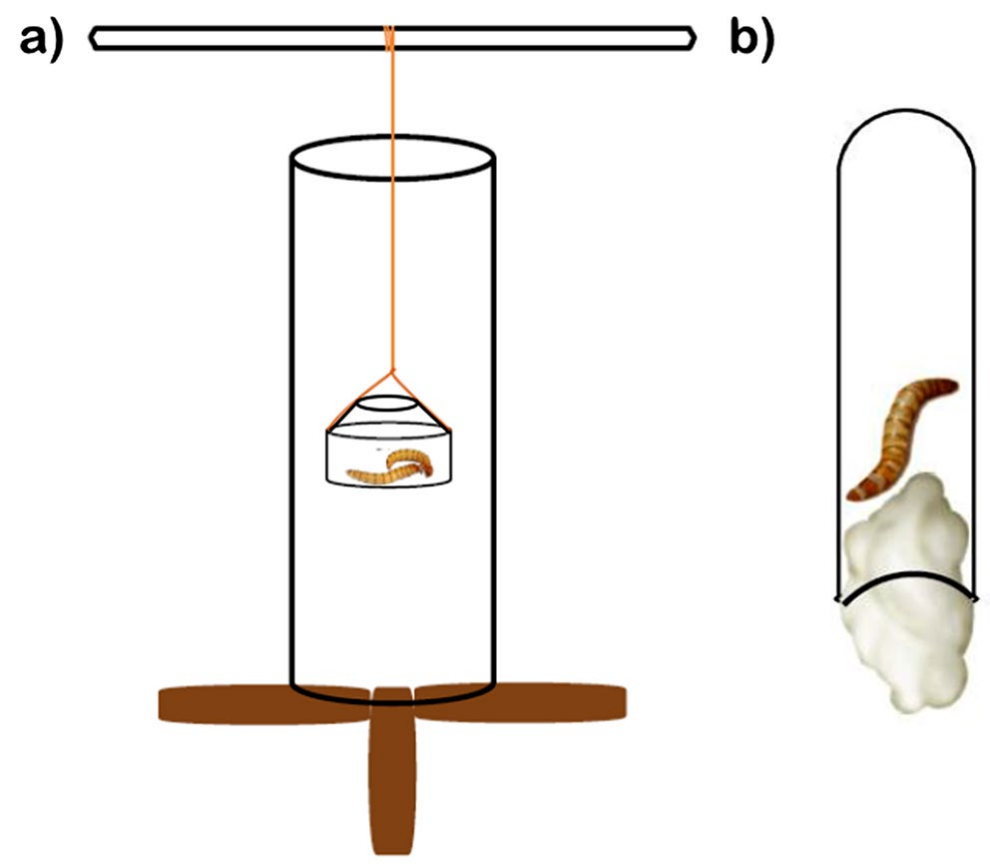
Schematic drawing of the test devices, not to scale (**a**: string-pulling, **b**: plug-opening)

Our test device consisted of a small (35 mm diameter) petri dish (with a bottleneck-like plastic rim attached to it to reduce the risk of the reward accidentally falling out) attached like a hanging bucket to a 17 cm string, hanging inside a vertically positioned transparent plastic tube with the opening facing upwards (Fig. 1a). In the dish, we had placed the food reward (two mealworms in 2015, reduced to one mealworm after it seemed sufficient from 2016 onward) that was visible but not directly accessible to the bird until it pulled up the string. We discarded the tests from three birds (two rural males and one rural female) in 2015 because they were presented with a test prototype where they had no plastic tube around the string. The remaining 27 birds from 2015 to 2017 had the string hanging into a thin-walled plastic tube crafted from plastic cups and stabilised with a wooden frame. The 36 birds in 2021–2022 had a sturdier plastic tube (150 mm tall and 70 mm wide, with a 3 mm thick wall), mounted on an upside-down ceramic dish, around the string; in 2022, a thinner rim was added to this sturdy tube.

We considered a session as solved when a bird pulled up the string and took out the worm from the dish. Out of the 252 trials of 63 birds included in our analyses, 16 had to be terminated early because the mealworm fell out of the dish before the bird could pull up the string (in 12 cases because the bird was shaking the string, and in four cases because the worm crawled past the rim of the dish). These were counted as unsuccessful tests, and in the analyses these birds were given maximal latencies. In eight trials, the birds successfully pulled up the string but lost the worm, dropping it back into the tube. These were counted as successful despite the fact that they could not get the prey, because the birds still went through the right set of motions to get the prey. In five trials the birds pulled up the string, dropped it outside the tube, and took the worm from the hanging dish; in one trial the bird stretched downward to reach all the way down to the rim of the dish and pulled it up before taking the worm out. Although these were both unconventional solutions, we still counted them as successful because the bird pulled up the dish in some innovative way. However, in four trials, the bird dived into the tube and ate the worm while in there, then attempted to get out. These trials were counted as unsuccessful despite the bird getting the worm, because this “solution” did not require innovation; three out of four of these birds managed to solve the problem in the conventional way afterwards.

### Plug-opening

In this test, we placed a mealworm inside a transparent tube that was closed by a cotton plug at its bottom end (Fig. 1b). In 2015–2017 and 2022, we used a 75 mm long and 11 mm wide glass tube; in 2021 it was a slightly larger, 100 mm long and 15 mm wide plastic tube. At the start of each session, we introduced this test device to a bird’s home cage attached to the cage wall next to a perch. In Groups 2015 and 2022, the tube’s bottom was 41 cm above the cage’s floor, whereas in 2016–2017 and 2021 it was only 26 cm above the cage floor. If a bird removed the plug, the mealworm would fall to the bottom of the cage and become accessible to the bird.

We considered a test successful when the bird removed the plug so that the worm fell out. Out of the 264 trials of 66 birds, there were six trials where the bird pulled out the cotton plug but lost the worm before eating it: in five trials it fell outside the cage and in one the bird could not find it in the cotton. In a seventh trial, the bird pulled out the cotton, but the worm got stuck in the tube. We counted these trials as successful despite the birds not getting the food reward. In 6 trials, the bird, instead of pulling the cotton with its beak, grabbed it with its foot and pulled it out. These solutions were also counted as successful. However, in one trial, the bird pulled out the cotton with its foot clearly by accident, as it did not pay attention to the tube and did not eat the worm afterwards; this trial was counted as unsuccessful. In two trials, the cotton fell out of the tube without the bird touching it, and in two other trials, the worm escaped from the tube, squeezing by the cotton plug, before the bird could solve the task. These trials were also counted as unsuccessful.

### Statistical analyses

For each task, we quantified problem-solving latency as the time (in seconds) from the start of the test until the bird solved the problem (took out the worm from the dish in the string-pulling test, pulled out the plug so that the worm fell out in the plug-opening test). We decided to use the start of the test rather than the first interaction with the test device because the bird was in a small enclosed space and could inspect the feeder already before interacting with it. For the unsuccessful sessions, we assigned a maximal latency value of 1201 s, even if they had to be terminated early due to the bird losing the worm. We assigned a separate latency value for each of the four tests of the same type, therefore each bird was included in the models with four trials.

We ran all our statistical analyses in R (version 3.6.1). We analysed problem-solving latency with Cox mixed-effects proportional hazard models, separate models for string-pulling and plug-opening, using the “coxme” R package (Therneau 2012). Survival models like the Cox proportional hazard model simultaneously handle variation in the probability of an event (such as solving success) and variation in latencies, making them well-suited for analysing behavioural latency data when there are individuals who do not show the focal behaviour (e.g. solve the task), as long as the proportional hazard requirement is met (Jahn-Eimermacher et al. 2011; Andersen et al. 2021). Therefore, they are often used in problem-solving studies (e.g. Cook et al. 2017; Preiszner et al. 2017; Prasher et al. 2019). In these models, we used problem-solving latency as the response variable, treating tests with maximal latencies (i.e. tests where the bird did not solve the task) as censored data. We included the following explanatory variables in our model: sessions number (1 to 4 for the four consecutive test sessions of the same type on the same individual) as a covariate, and habitat type (urban vs. rural), sex (male vs. female), age (first-year vs. older) and year (four levels: 2015, 2016–2017, 2021 and 2022) as factors. The variable “year” also controls for the identity of the experimenter, as it was always the same person within a year but a different person each year except in 2021 and 2022. We treated 2021 and 2022 as separate years because we implemented changes in the methods between December 2021 and January 2022 (see above), whereas 2016–2017 was treated as a single year because there were no such changes in the protocol. We also included bird ID nested within capture site as random factors to control for autocorrelation within individual and within population, respectively. We henceforth refer to these models as ‘base models’. As stepwise model selection based on p-values, despite being frequently used, is also often criticized (Garamszegi et al. 2009), we opted to present the estimates both from the full base models and from reduced models where explanatory variables with P-values over 0.1 were eliminated. We refer to explanatory variables with P-values below 0.05 as “statistically significant” and those with P-values between 0.05 and 0.1 as “tendencies” or “trends”. For pairwise comparisons between the four years, we extracted parameter estimates by using the ‘emmeans’ function of the ‘emmeans’ R package (Lenth et al. 2019). We present both the unadjusted P-values, as these were identical to the P-values from the model summary; and P-values adjusted for comparing a family of four estimates by Tukey’s method (Tukey 1949) using a function built into the ‘emmeans’ package.

Neophobia could not have been quantified for one for one individual (the one that did not take the food item in the control phase of the neophobia test, see the relevant part in Methods). Therefore, including neophobia in the base model would have meant excluding this individual from our analyses altogether, which would have led to reduced sample sizes and data loss. Instead, we built extended models (separately for plug-opening and for string-pulling) which were identical to our base models except also including log-transformed neophobia as a covariate, and we report the parameter estimate for neophobia from these extended models. The other variables in the extended models, despite the reduced sample size, yielded estimates qualitatively similar to the base models. To avoid multicollinearity, we also tested whether neophobia was affected by any of our tested factors in a single linear model with habitat type, sex, age and group as covariates; none of these variables had a significant effect on neophobia (Table S2).

We tested whether learning speed (i.e. the change of latencies over the four sessions, included in the model as the variable “session number”) differed between habitat types, sexes and age groups by adding interaction terms between session number × habitat type, session number × sex or session number × age to our models. To avoid over-parametrization, we used the same approach as with neophobia, by adding only one interaction term at a time to the base models (including both statistically significant and non-significant terms), and reporting only the interaction estimates. The variables not included in the interaction in these extended models yielded qualitatively similar results to the base model.

To test whether the solving latencies in the two tests were correlated with each other, we used the same modelling approach as with neophobia and the interactions: we built extended models identical to our base models except also including solving latency in the other type of problem-solving test (string-pulling latency’s effect on plug-opening latency and vice-versa) as a covariate. This covariate was log-transformed, and non-solvers were given maximal latency values of log(1201) = 7.091. Sessions were paired by session number, i.e. the first string-pulling test with the first plug-opening test, the second string-pulling with the second plug-opening, and so forth. As with the above models, we only report the parameter estimate for this variable from these extended models; the other variables yielded estimates qualitatively similar to the base models. We report both string-pulling latency’s effect on plug-opening latency and plug-opening latency’s effect on string-pulling latency despite the fact that these test the same hypothesis, as there is no biological or mathematical justification to choose one model over the other.

We could not include the variable “test order” in our models due to its multicollinearity with the variable “year”. However, we tested its effect by building Cox models identical to our base model except replacing the variable “year” with the variable “test order” and excluding the birds from 2016 to 2017. This variable had no significant effect on either string-pulling latency (coefficient ± SE = -0.377 ± 0.627; Z = -0.600; *P* = 0.550) or plug-opening latency (coefficient ± SE = 1.032 ± 0.719; Z = 1.440; *P* = 0.150).

## Results

### String-pulling

**Fig. 2:**
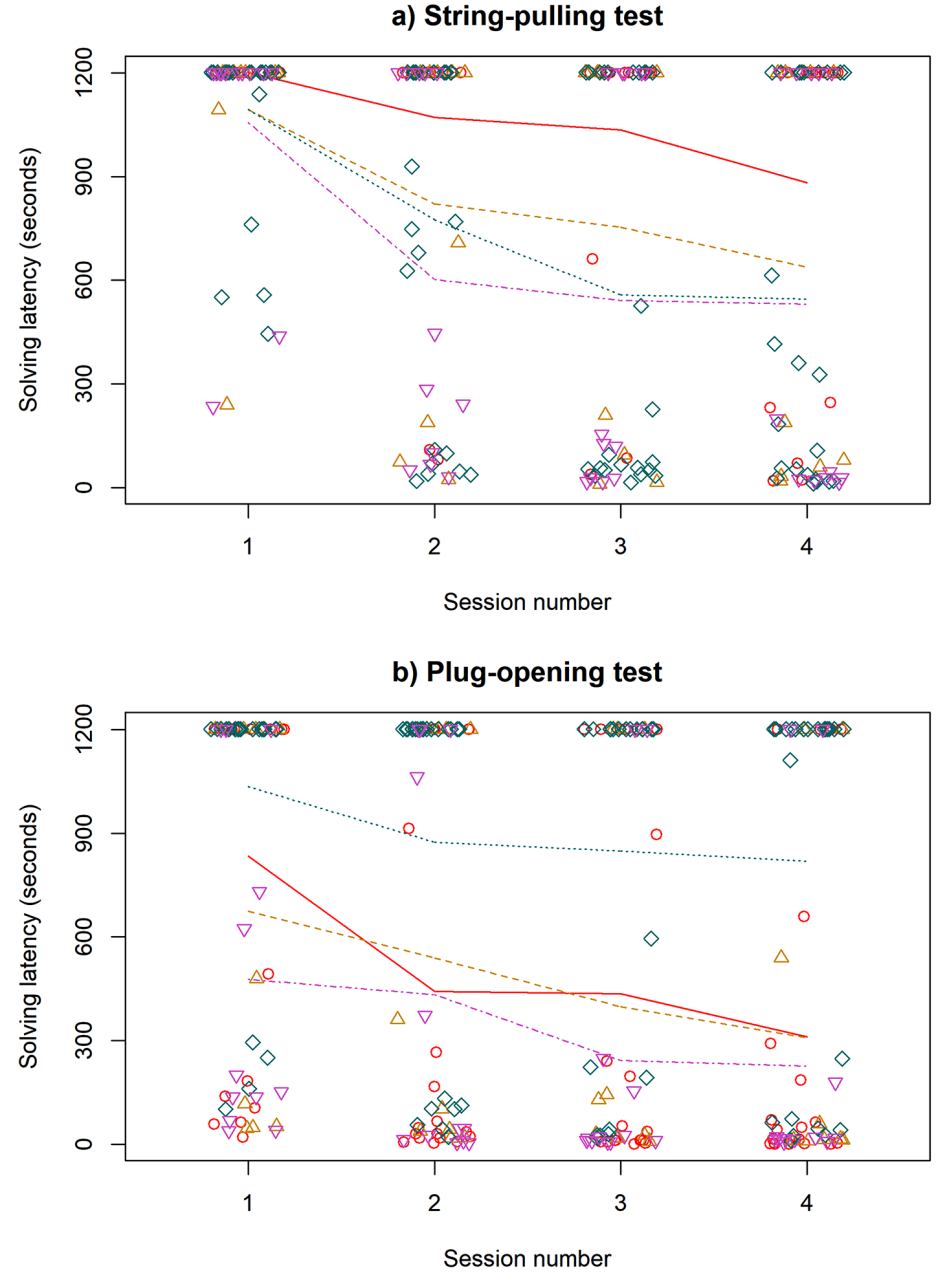
Solving latencies in the four sessions of (**a**) string-pulling tests and **b**) plug-opening tests. Unsuccessful birds are represented with a latency of 1201 s. The data points are scattered horizontally for visibility. The lines represent the mean learning curves for each of the four years, connecting the mean solving latencies of each yearly cohort in each session. The color and the shape of the data points and lines represents the four experimental years (2015: red circles, solid line; 2016–2017: golden upward triangles, dashed line; 2021: teal diamonds, dotted line; 2022: purple downward triangles, dash-dotted line)

Overall, this test was solved by 34 out of 63 birds (54.0%) at least once; there were 9 successful solutions (14.3%) in the first, 25 (39.7%, including successful solutions by birds who solved in the previous sessions; 16 first solutions) in the second, 28 (44.4%; 5 first solutions) in the third and 32 (50.8%; 4 first solutions) in the fourth session. According to the full Cox model (Table 2a), solving latencies on average decreased over the four sessions, and females tended to solve faster than males, whereas there was no significant difference between either urban and rural or adult and juvenile birds. Furthermore, the birds in 2021 and 2022 solved the problem significantly faster than the birds in 2015, with the 2016–2017 birds having an intermediate value not significantly different from the other three (Table 2a, Fig. 2a). Birds from 2015 were less likely to solve (5 out of 17 birds, 29.4%) than those from 2021 (15 out of 24, 62.5%), 2022 (9 out of 12, 75.0%) and tendentially 2016–2017 (5 out of 10; 50.0%). After removing the non-significant effects of environment and age, the effect of session number (coef ± SE = 1.167 ± 0.127; Z = 9.180; *P* < 0.001), sex (coef ± SE = -1.730 ± 0.922; Z = -1.877; *P* = 0.061) and year (2021 vs. 2015: coef ± SE = 2.444 ± 1.114; Z = 2.195; *P* = 0.028; 2022 vs. 2015: coef ± SE = 2.411 ± 1.214; Z = 1.705; *P* = 0.088; unadjusted P-values) remained qualitatively similar. Neophobia had no significant effect on solving latency (coef ± SE = 0.405 ± 0.791; Z = 0.510; *P* = 0.610). The interactions showed that learning speed (i.e. the effect of test number on solving latency) did not differ between urban and rural (coef ± SE = 0.182 ± 0.217; Z = 0.840; *P* = 0.400), male and female (coef ± SE = -0.123 ± 0.223; Z = -0.550; *P* = 0.580), or juvenile and adult birds (coef ± SE = 0.155 ± 0.209; Z = 0.550; *P* = 0.580).

**Table 2:** Effects of our explanatory variables on problem-solving latency in the string-pulling test (a) and the plug-opening test (b), extracted from summary tables of our Cox mixed-effects models. Pairwise comparisons between years are estimated marginal means; for these comparisons we provide both unadjusted P-values (extracted directly from the model summary) and P-values adjusted for comparison between 4 classes with Tukey’s method. More positive values indicate increasingly faster solving (i.e. shorter latencies) for covariates (session number) and faster solving by the compared level (listed first) than the reference level (listed second) for factors. Statistically significant effects (*P* < 0.05) are marked in bold; statistically non-significant trends (0.05 < *P* < 0.10) are marked in bold italic

a) String-pulling					
Fixed effects	Coefficient	± SE	Z	*P*_unadj_	*P*_Tukey_
Session number	**1.120**	**± 0.123**	**9.090**	**< 0.001**	
Environment (urban vs. rural)	-1.042	**±** 0.769	-1.354	0.180	
Sex (male vs. female)	***-1.404***	***± 0.771***	***-1.822***	***0.068***	
Age (juvenile vs. adult)	0.742	± 0.838	0.886	0.380	
Year (2016–2017 vs. 2015)	1.772	± 1.207	1.468	0.142	0.457
Year (2021 vs. 2015)	**2.185**	**± 0.908**	**2.406**	**0.016**	***0.076***
Year (2022 vs. 2015)	**2.289**	**± 1.095**	**2.089**	**0.036**	0.157
Year (2021 vs. 2016–2017)	0.414	**±** 1.042	0.326	0.717	0.984
Year (2022 vs. 2016–2017)	0.517	**±** 1.304	0.392	0.692	0.979
Year (2022 vs. 2021)	0.103	**±** 1.047	0.099	0.921	0.999
**Random effects**	**SD**				
Site	0.283				
Bird ID nested in Site	2.128				

b) Plug-opening					
Fixed effects	Coefficient	± SE	Z	*P*_unadj_	*P*_Tukey_
Session number	**0.778**	**± 0.093**	**8.370**	**< 0.001**	
Environment (urban vs. rural)	-0.158	± 0.594	-0.266	0.791	
Sex (male vs. female)	-0.266	± 0.611	-0.434	0.664	
Age (juvenile vs. adult)	0.456	± 0.661	0.690	0.490	
Year (2016–2017 vs. 2015)	0.131	± 0.861	0.152	0.879	0.999
Year (2021 vs. 2015)	**-2.158**	**± 0.707**	**-3.052**	**0.002**	**0.012**
Year (2022 vs. 2015)	0.584	± 0.851	0.686	0.493	0.903
Year (2021 vs. 2016–2017)	**-2.289**	**± 0.836**	**-2.739**	**0.006**	**0.031**
Year (2022 vs. 2016–2017)	0.453	± 1.026	0.441	0.659	0.971
Year (2022 vs. 2021)	**2.741**	**± 0.817**	**3.356**	**0.001**	**0.004**
**Random effects**	**SD**				
Site	0.192				
Bird ID nested in Site	1.835				

### Plug-opening

Altogether, 46 out of 66 birds (69.7%) solved the task at least once; there were 25 successful solutions (37.9%) in the first, 36 (54.5%, including successful solutions by birds who solved in the previous sessions; 13 first solutions) in the second, 39 (59.1%; 5 first solutions) in the third and 43 (65.2%; 3 first solutions) in the fourth session. Our full Cox model showed that solving latencies decreased over the four sessions, and there was no significant difference between urban and rural, male and female, or adult and juvenile birds (Table 2b). Year had a significant effect: birds in 2021 solved significantly slower than from the other years, which were not significantly different from one another (Table 2b; Fig. 2b). Birds from 2021 were less likely to solve (10 out of 24 birds, 41.7%) than those from 2015 (16 out of 20, 80%), 2016–2017 (8 out of 10, 80%) and 2022 (12 out of 12 birds, 100%). After removing the non-significant effects of environment, sex and age, the effect of session number (coef ± SE = 0.881 ± 0.093; Z = 8.390; *P* < 0.001) and year (2021 vs. 2015: coef ± SE = -2.105 ± 0.709; Z = -2.969; *P* = 0.003; 2021 vs. 2016–2017: coef ± SE = -2.163 ± 0.848; Z = -2.551; *P* = 0.011; 2021 vs. 2022: coef ± SE = -2.737 ± 0.807; Z = -3.391; *P* = 0.001; unadjusted p-values) remained qualitatively the same. Neophobia had no statistically significant effect (coef ± SE = -0.041 ± 0.596; Z = -0.070; *P* = 0.940). The interactions showed that learning speed (i.e. the effect of test number on problem-solving latency) did not differ between urban and rural (coef ± SE = 0.051 ± 0.166; Z = 0.310; *P* = 0.760), male and female (coef ± SE = 0.100 ± 0.167; Z = 0.600; *P* = 0.550), or juvenile and adult birds (coef ± SE = -0.197 ± 0.172; Z = -1.150; *P* = 0.250).

### Relationship between the two tests

**Fig. 3:**
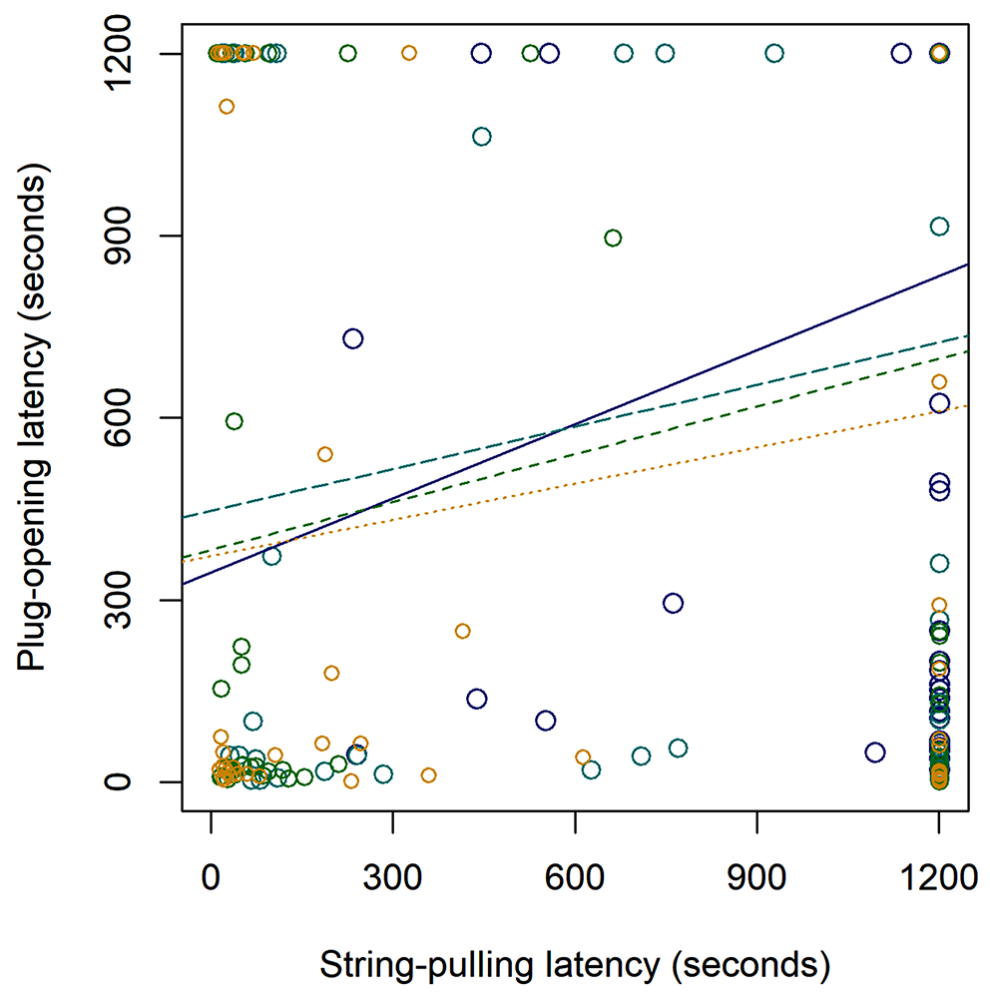
Relationship between solving latencies in the plug-opening and string-pulling tests; each dot represents a latency of a bird in the first, second, third and fourth session. Later sessions are represented by warmer colors, smaller dots and lines with shorter dashes. The lines represent the relationship between the solving latencies in the two test types for each of the four consecutive sessions, fitted using linear regression with plug-opening latency as the response variable and string-pulling latency as covariate

We found a positive relationship between solving latencies in the two tests, which was statistically significant when plug-opening was the response variable (coefficient ± SE = -0.226 ± 0.112; Z = -2.010; *P* = 0.045), but only a trend when string-pulling was the response variable (coefficient ± SE = -0.213 ± 0.112; Z = -1.910; *P* = 0.057; Fig. 3); note that because we used Cox models, negative coefficients indicate positive relationship (i.e. birds that solved one test faster also had lower latencies in the other test). Out of the 63 birds that participated in both tests, 27 solved both tests at least once; 7 solved the string-pulling but not the plug-opening; 16 solved the plug-opening but not the string-pulling; and 13 did not solve either test.

## Discussion

In our study, we investigated the problem-solving abilities of urban and rural great tits in a string-pulling and a plug-opening task. We found higher problem-solving success in the string-pulling test compared to earlier studies, and decreasing latencies over repeated sessions in both task types, indicating individual learning, as well as a weak relationship between solving successes and latencies in the two tasks. Furthermore, we did not find any significant difference in either mean solving latencies or the decrease of solving latencies over repeated sessions between urban and rural birds, between juveniles and adults, and only a slight difference in the string-pulling task between males and females. However, we found differences between the solving latencies of birds from different years in both tasks, which is likely due to methodological factors (summarized in Table 1), and therefore highlights the issue of replicability of studies. We discuss these results below.

### String-pulling success and latency

Interestingly, the percentage of successful birds in the string-pulling test was higher than in previous string-pulling tests in this species (Thorpe 1956; Vince 1956; Cole et al. 2011). Vince (1956) performed the test on 12 birds, out of which only 1 solved the problem upon the first trial (8.3% solving success). Thorpe (1956) found that 4 out of 28 birds (14.3%) managed to pull up the string, whereas Cole et al. (2011), with slightly different experimental set-up, found that 93 out of their 365 birds (25.5%) solved the test successfully. In our study, in the first 20-minute trial, 14.3% of our birds solved the test, a very similar ratio to what Thorpe (1956) found; however, by the end of the fourth 20-minute trial, 54.0% solved the string-pulling task at least once, double of the 25.5% that Cole et al. (2011) found. The difference between ours and previous results is greater if we look at the years separately: in 2015, only 29.4% of the birds solved the task at least once, which is only slightly above what Cole et al. have found, but by 2022, this numer increased to 75.0%.

Several factors can explain these relatively high solving success rates. First, our birds may have been more acclimatised to captive conditions. Rather than testing the birds only a day after capture like Cole et al. (2011), our birds had been in captivity for at least three days before the first session of the problem-solving tests. This extra time may have helped the birds become less stressed when interacting with the test device (but see Butler et al. 2006). Second, our birds may have been more motivated than those in previous studies. Motivation plays an important role in problem-solving success (Horik and Madden 2016). Unlike Vince (1956), who baited the test device with seeds, Cole et al. (2011) and we both baited it with more attractive live insect prey (waxworms or mealworms). Instead of fasting the birds like Vince (1956) and Cole et al. (2011) did, we motivated them by keeping them on a seed diet prior to the experiments. Great tits are better at problem-solving tasks with live insect food as the reward when kept on a seed diet than when they are kept on an insect diet (Davidson et al. 2020). Third, rather than participating in a single, up to an hour-long trial like the birds in the previous studies (Vince 1956; Cole et al. 2011), our birds’ learning time was broken down to four relatively short (up to 20 min long) sessions. Usually, great tits will inspect new items quickly and then lose interest after a few unsuccessful attempts (Johnsson and Brodin 2019). Therefore, it is possible that our birds, after losing interest upon failing to solve for the first time, regained their motivation by the time of the next session. This idea is supported by the fact that 23 of our 34 successful first solutions (67.6%) happened in the first half of the successful session (median solving latency in this subset: 361 s).

### Plug-opening success and latency

The plug-opening task has never been performed on great tits, but multiple times on mountain chickadees (Kozlovsky et al. 2015, 2017). While most birds eventually solved the task in these studies, it usually took them several one-hour sessions. Compared to this, our great tits solved much faster, with 38% of them figuring out the solution by the end of the first 20-minute trial, and 70% of them having solved at least once by the fourth 20-minute trial. This could be a difference between species and could be explained by the differences in their ecology: mountain chickadees are specialized food-hoarders, making them specialized in spatial memory tasks (Croston et al. 2016; Sonnenberg et al. 2019), whereas, great tits are non-hoarding opportunists that benefit more from innovativeness and exploiting novel food sources compared to other parid species (Sasvári 1979; Urhan et al. in prep.). Alternatively, it is possible that the fast plug-opening success of our great tits can be explained by a carry-over effect from the string-pulling test that, for the majority of our birds, directly preceded the plug-opening test. However, this is unlikely, because test order had no significant effect on solving latency in the plug-opening test.

### Learning effect

After the birds successfully solved one task, most of them consistently solved it afterwards, with their solving latency becoming shorter with each consecutive session. This suggests that the birds were learning over the four trials, memorizing the correct solution and improving with experience. Interestingly, even the birds tested in 2016–2017 showed this learning pattern, despite the fact that there were sometimes longer gaps (up to 12 days) between two trials of the same type. There were only three birds that failed to solve a string-pulling session after being successful in a previous session, due to trying to pull the string too vigorously and shaking the worm out. Similarly, there were three birds that lost the food reward after successfully solving the plug-opening test, and then did not solve the task in consecutive trials; presumably, losing the worm served as negative reinforcement in these cases. Other studies on the string-pulling ability of great tits found similar learning patterns. When Cole et al. (2011) recaptured 47 birds and performed the string-pulling test on them for a second time, 27 birds (57.4%) were successful. Vince (1956) trained some of the unsuccessful birds with strings of increasing length; this training was successful for 4 out of 9 individuals (44.4%). These results all support the idea that in great tits, learning abilities affect problem-solving performance (Cauchard et al. 2024).

### Relationship between the two tests

We found a tendency that birds that solved the string-pulling task faster also had shorter solving latencies in the plug-opening task. This can be explained by among-individual variation in general (rather than task-specific) problem-solving ability or overall cognitive ability, or a carry-over effect from one task type to another (i.e. solving success in one task type facilitates solving success in the other task). Our finding is similar to Cole et al. (2011), who found a relationship between solving success in the string-pulling task and a cognitive test in which the great tits could access a food item on a platform by pulling a lever. By contrast, Preiszner et al. (2017) found no correlation between solving success in an obstacle-removal task and a lid-opening food acquisition task. This lack of correlation may be due the very different rewards in the two tests (food items versus access to nestlings) and thus had very different motivational drives, unlike Cole et al. (2011) and our study where the reward was food in all tests.

### Effects of urbanization

Our initial prediction that urban individuals will be better problem-solvers than their rural conspecifics was not supported by our results. This appears to be contrary to a number of studies that found that urban animals perform better than rural animals in cognitive tasks (Liker and Bókony 2009; Sol et al. 2011; Audet et al. 2016; Kozlovsky et al. 2017; Solaro et al. 2019; Chow et al. 2021; Mazza and Guenther 2021), including two other studies on great tits (Preiszner et al. 2017; Grunst et al. 2020). A possible explanation for this discrepancy is that the latter two studies were performed on breeding pairs in the wild, whereas we performed our study indoors on wild-caught birds. Animals in captivity may perform either better (Morand-Ferron et al. 2011; Benson-Amram et al. 2013) or worse (McCune et al. 2019) in cognitive tasks compared to their wild conspecifics. Indoor environment is more standardized and easier to control, which may remove differences due to environmental conditions. For example, in forest habitats, a high abundance of insects may decrease the birds’ motivation to solve a food extraction task compared to urban habitats with lower insect abundance (Preiszner et al. 2017), whereas our captive birds were kept on an identical seed diet in order to make them equally motivated.

Alternatively, the lack of urban-rural differences in our and some other studies (Papp et al. 2015; Cook et al. 2017; Morton et al. 2023) and the better performance of rural animals in a few others (Prasher et al. 2019; Johnson Ulrich et al. 2021) can be explained by the poor nutritional conditions (Seress et al. 2018) and other forms of environmental stress and that has been suggested to occur in urban habitats (Birnie-Gauvin et al. 2016). Physiological condition and stress both can affect problem-solving performance (Bókony et al. 2014; Cook et al. 2017; but see Grunst et al. 2020), counteracting the stronger necessity for cognitive abilities to cope with such habitats.

### Differences between years

In both problem-solving tasks, the factor with the strongest effect was the year in which the experiment was performed. Besides a temporal effect, this variable also encompasses an observer effect (one observer in 2015, another one in 2016–2017 and a third one in 2021 and 2022) and differences in methodology (Table 1, Supplementary material 1). String-pulling success was higher in each following year than the previous one. While it is theoretically possible that our study populations have gradually become better at the string-pulling task over the course of the years, it is rather unlikely that they would have encountered problems similar to the string-pulling test in their natural habitats. Alternatively, the between-year differences could be explained by differences between the environmental (e.g. weather) conditions that the birds experienced before getting captured.

However, the most likely explanation is that problem-solving success was affected by the slight differences in experimental methodology between years. For example, the birds could solve the string-pulling task easier if they could perch on the edge of the plastic tube into which the string was hanging rather than the perch to which the string was tied. In 2015–2017, the string was hanging into a thin-walled plastic tube made from two plastic cups and mounted on a wooden frame, which was somewhat unstable and difficult to perch on. By contrast, in 2021–2022, it was hanging within a sturdy plastic cylinder, firmly mounted on a ceramic dish, providing a rather stable surface the bird could perch on and hold the string to (even after we added a thinner plastic rim in 2022), explaining their greater problem-solving success.

In the plug-opening task, the birds from 2021 had much worse problem-solving success (42%) than from 2015 (80%), 2016–2017 (80%) or 2022 (100%). There were several differences in the experimental methods potentially explaining this between-year variation. In 2021, the plugged tube was somewhat bigger and made of plastic, whereas in the other years it was a slightly smaller tube made of glass. This could have affected the visibility of the mealworm and thus the motivation of the birds, as well as the size and the resistance of the cotton plug and thus the effort required to solve it. Furthermore, in 2015 and 2022 all perches other than the one next to the plugged tube were removed, therefore the birds were physically forced to spend time in the proximity of the device, whereas in 2021, there were other perches in the cage, providing more opportunities for the birds to not to interact with the device. However, other perches were also available in 2016–2017, meaning that this latter methodological mismatch, by itself, cannot explain all observed differences between years.

### Replicability and comparability between studies

The differences in solving success depending on small differences in our lab highlight a more general problem about the replicability of behavioural experiments. Replicability is an important measure of the reliability of scientific studies, yet behavioural experiments are seldom repeated, and when they are, they often yield different results (Brecht et al. 2021; Farrar et al. 2021). It is important to differentiate between conceptual and direct replications. The former is when the same research question and hypothesis are tested with different methods. Despite the variation in methodology, these studies are often used for within- and between-species comparisons (Kabadayi et al. 2016, 2017; Isaksson et al. 2018), and often form the basis of meta-analyses. It is perhaps unsurprising that these comparisons are often inconclusive: for example, Vincze and Kovács (2022) found large heterogeneity in their meta-analysis of studies comparing cognition of urban and rural conspecifics, but it is unclear whether that is due to differences between study species or differences between methodologies.

By contrast, a direct replication is when a study tries to replicate an earlier study’s exact experimental methods. In our case, reprising the 2015–2017 experiment in 2021–2022 could be seen as an attempt at a direct replication. These are uncommon, partly because they lack novelty, which reduces their publishability (Brecht et al. 2021), and partly because they are often difficult to perform, especially across labs but sometimes even within the same lab. This is often due to logistic constraints (e.g. it is difficult to sample the exact same populations, or the exact same equipment is not available) or inadequate communication (e.g. the methods are not described in enough detail). The differences we found between years in both problem-solving tests suggest that fine methodological details which are often overlooked, such as the size and material of the test devices, can affect the results.

On the other hand, slight changes to the experimental protocol can also work as a refinement of the methods. Regarding the string-pulling test, we improved upon the methods used by Vince (1956), Thorpe (1956) and Cole et al. (2011), at the same time as we tried to improve the experimental protocol over the years when we encountered problems. The increase in problem-solving success across years that we found in the string-pulling test indicates that we managed to improve the protocol of this particular test. This suggests that the cognitive performance of animals may be underestimated due to experimental methods less suitable for the species.

Overall, the strong difference between study years, despite our effort to keep the methodology consistent across years and experimenters, has an important implication: if experiments on the same populations with only small differences in methodology can yield such different results, then studies performed by different researchers at different labs, on different species or different populations of the same species, at different geographical locations, using non-identical experimental protocols must be even harder to compare. Therefore, we need to always be careful when drawing conclusions from such comparisons between studies.

Furthermore, we would like to emphasize the importance of a detailed description of the experimental protocol, and advise other authors not to exclude certain details from their methods description just because they are subjectively assumed to be irrelevant. In this digital age, video recordings are easy to make and share, which can be a helpful visual aid when replicating experiments. These methodological details must not be overlooked if we want to make meaningful generalizations and between- and within-species comparisons.

## Electronic supplementary material

Below is the link to the electronic supplementary material.

Supplementary Material 1

Supplementary Material 2

Supplementary Material 3

## Data Availability

Data is provided within the supplementary material files (ESM 2).
