# Supplementary material for: Are comparable studies really comparable? Suggestions from a problem-solving experiment on urban and rural great tits
Source: Anim Cogn. 2024 Jul 9;27(1):47. doi: 10.1007/s10071-024-01885-3 (PMC11233327; doi:10.1007/s10071-024-01885-3)
Supplement: Supplementary file 2 — Supplementary Material 2 [file 10071_2024_1885_MOESM2_ESM.docx]

**Are comparable studies really comparable? Suggestions from a problem-solving experiment on urban and rural great tits**

Ernő Vincze, Ineta Kačergytė, Juliane Gaviraghi Mussoi, Utku Urhan, Anders Brodin

**Online Resource 2: Supplementary Tables**

**Table S1:** Study sites with habitat type (rural or urban), the region the site is part of, and the number of birds of each age (A: adult, J: juvenile) and sex (M: male, F: female) combination captured from each site.

| **Site name** | **Habitat type** | **Region** | **AM** | **AF** | **JM** | **JF** |
| --- | --- | --- | --- | --- | --- | --- |
| Backen | Rural | Höör | 1 | 2 | 0 | 0 |
| Gäddangen | Rural | Höör | 3 | 1 | 2 | 2 |
| Karlsund | Rural | Höör | 1 | 2 | 0 | 1 |
| Linekulsvagen | Rural | Höör | 3 | 2 | 0 | 0 |
| Ormapumpan | Rural | Höör | 4 | 0 | 0 | 2 |
| Orups sjukhus | Rural | Höör | 2 | 1 | 0 | 0 |
| Vaxsjön | Rural | Höör | 0 | 3 | 0 | 0 |
| Stensoffa | Rural | Stensoffa | 0 | 0 | 2 | 0 |
| Ekologihuset | Urban | Lund | 8 | 2 | 3 | 4 |
| Linero | Urban | Lund | 5 | 4 | 0 | 3 |
| Malmö | Urban | Malmö | 0 | 1 | 0 | 2 |

**Table S2:** Estimates from the summary of a linear model testing the effects of environment, sex, age and year on neophobia (log-transformed). Pairwise comparisons between years are calculated as contrasts between estimated marginal means. DF = 58 for all comparisons. The lack of statistically significant effects indicates that neophobia does not show multicollinearity with the other variables in our models.

| **Fixed effects** | **Estimate** | **± SE** | **t** | **P** |
| --- | --- | --- | --- | --- |
| Intercept | 6.149 | ± 0.161 | 38.243 | <0.001 |
| Environment (urban vs rural) | 0.065 | ± 0.132 | 0.489 | 0.627 |
| Sex (male vs female) | -0.042 | ± 0.140 | -0.300 | 0.765 |
| Age (juvenile vs adult) | -0.029 | ± 0.150 | -0.194 | 0.847 |
| Year (2016-2017 vs 2015) | -0.117 | ± 0.205 | -0.573 | 0.569 |
| Year (2021 vs 2015) | 0.044 | ± 0.163 | 0.272 | 0.787 |
| Year (2022 vs 2015) | 0.036 | ± 0.194 | 0.188 | 0.852 |
| Year (2021 vs 2016-2017) | 0.161 | ± 0.202 | 0.799 | 0.427 |
| Year (2022 vs 2016-2017) | 0.154 | ± 0.228 | 0.673 | 0.503 |
| Year (2022 vs 2021) | -0.008 | ± 0.195 | -0.041 | 0.967 |
